# Fungal Infection Induces Anthocyanin Biosynthesis and Changes in DNA Methylation Configuration of Blood Orange [*Citrus sinensis* L. (Osbeck)]

**DOI:** 10.3390/plants10020244

**Published:** 2021-01-27

**Authors:** Angelo Sicilia, Vittoria Catara, Emanuele Scialò, Angela Roberta Lo Piero

**Affiliations:** Department of Agriculture, Food and Environment (Di3A), University of Catania, Via Santa Sofia 98, 95123 Catania, Italy; angelo.sicilia@unict.it (A.S.); vcatara@unict.it (V.C.); escialo92@gmail.com (E.S.)

**Keywords:** *Citrus sinensis*, sweet orange, anthocyanin, DNA methylation, gene expression, biotic stress, *Penicillium digitatum*

## Abstract

The biosynthesis of sweet orange anthocyanins is triggered by several environmental factors such as low temperature. Much less is known about the effect of biotic stress on anthocyanin production in sweet orange, although in other species anthocyanins are often indicated as “defense molecules”. In this work, citrus fruits were inoculated with *Penicillium digitatum*, the causal agent of green mold, and the amount of anthocyanins and the expression of genes related to their biosynthesis was monitored by RT-real time PCR after 3 and 5 days from inoculation (DPI). Moreover, the status of cytosine methylation of DFR and RUBY promoter regions was investigated by McrBC digestion followed in real-time. Our results highlight that fungal infection induces anthocyanin production by activating the expression of several genes in the biosynthetic pathway. The induction of gene expression is accompanied by maintenance of high levels of methylation at the DFR and RUBY promoters in the inoculated fruits, thus suggesting that DNA methylation is not a repressive mark of anthocyanin related gene expression in sweet orange subjected to biotic stress. Finally, by measuring the expression levels of the *Citrus* DNA demethylase genes, we found that none of them is up-regulated in response to fungal infection, this result being in accordance with the observed maintenance of high-level DFR and Ruby promoter regions methylation.

## 1. Introduction

The anthocyanins present in the red oranges have received great attention due to their contribution to the organoleptic qualities of fruits as well as to the beneficial health effects on either humans or animals [1]. The biochemical basis determining anthocyanin biosynthesis in orange fruits have been broadly studied and documented [2]. Multiple enzymes such as phenylalanine ammonia-lyase (PAL), chalcone synthase (CHS), chalcone isomerase (CHI), flavonoid 3′,5′-hydroxylase (F3′5′H), flavonoid 3′-hydroxylase (F3H), dihydroflavonol reductase (DFR), anthocyanidin synthase (ANS) and UDP-glucose flavonoid 3-O-glucosyltransferase (UFGT) constitute the metabolic pathway leading to pigment production (Figure S1) [3,4,5]. The MYB and bHLH transcriptional factor families finely control the switch on of this pathway [6]. More specifically, the pigmentation of blood oranges originates from a retrotransposon insertion that allows the expression of the *ruby* gene encoding the MYB-type transcription factor involved in the activation of the anthocyanin biosynthetic pathway [7]. In addition to the genetic nature of sweet orange blood germplasms, several abiotic environmental factors influence the pigmentation of fruits, such as light, nutritional status, xenobiotic or hormone treatments, and low temperature [8,9,10,11,12,13,14]. However, no studies have investigated the possible causal relationships between fruit-pathogen interaction and blood orange coloration. Flavonoids and anthocyanins are known to be involved in plant protection against pathogens [15]. It has been shown that purple tomato fruit, enriched in anthocyanin content by the ectopic expression of *Delila* and *Rosea1* genes encoding transcription factors, shows reduced susceptibility to *Botrytis cinerea*. This gained resistance depends specifically on the accumulation of anthocyanins, which can slacken the oxidative damage due to fungal infection [16]. Interestingly, mango fruit exposed to sunlight at the exterior of the canopy acquires a red peel color compared to the green peel fruit that develops in the shade [17]. The red tissues showed a significant increase in total anthocyanins and flavonoids and are associated with high tolerance to cold injury and more resistance to *Colletotrichum gloeosporioides* inoculation [17]. Epigenetic factors such as DNA methylation have emerged as relevant modulators of plant responses to the environment [18]. DNA methylation refers to the addition of a methyl group to the cytosine bases of DNA to form 5-methylcytosine that occurs predominantly on the CG, then CHG, and CHH context, respectively [19]. Most of the research works have been focused on the role of DNA methylation in determining plant phenotype in response to abiotic stress, whereas only a few studies have tried to shed the light upon how biotic interactions might affect DNA methylation configuration. It has been shown that virulent *Pseudomonas syringae* induces modifications of the DNA methylation status in the model plant *Arabidopsis thaliana* [20]. Methylation changes are frequently found in the proximity of defense-related genes and correlate with their transcriptional activation upon treatment, suggesting a role in the response to the pathogen [20]. *Penicillium digitatum*, the causal agent of green mold, represents the major postharvest pathogen of citrus fruits worldwide. During the postharvest period, it causes remarkable yield loss with a considerable detrimental economic impact [21,22]. A deeper understanding of the host-pathogen interactions is essential to clarify the functioning of molecular mechanisms underlying the infection and eventually to develop new methods for the storage, transport, and post-harvest marketing of citrus fruits. Global transcriptome analyses have been provided to identify genes specifically involved in *P. digitatum* interaction with citrus fruit [21,22]. However, those valuable studies have been performed upon blond orange varieties thus the lack of information upon the relation between anthocyanin production and the response to fungal infection remains. Consequently, in this work, we evaluated the effect of *P.digitatum* inoculation on the anthocyanin content and the expression of genes involved in their biosynthesis pathway after 3 and 5 days from inoculation (DPI). Moreover, the level of the *dfr* and *ruby* promoter DNA methylation was monitored to combine the gene expression results with the DNA methylation dynamics during fungal infection. In this respect, the expression level of DNA de-methylases involved in DNA methylation rearrangements was also measured.

## 2. Results

### 2.1. Fruit Appearance

**Figure 1:**
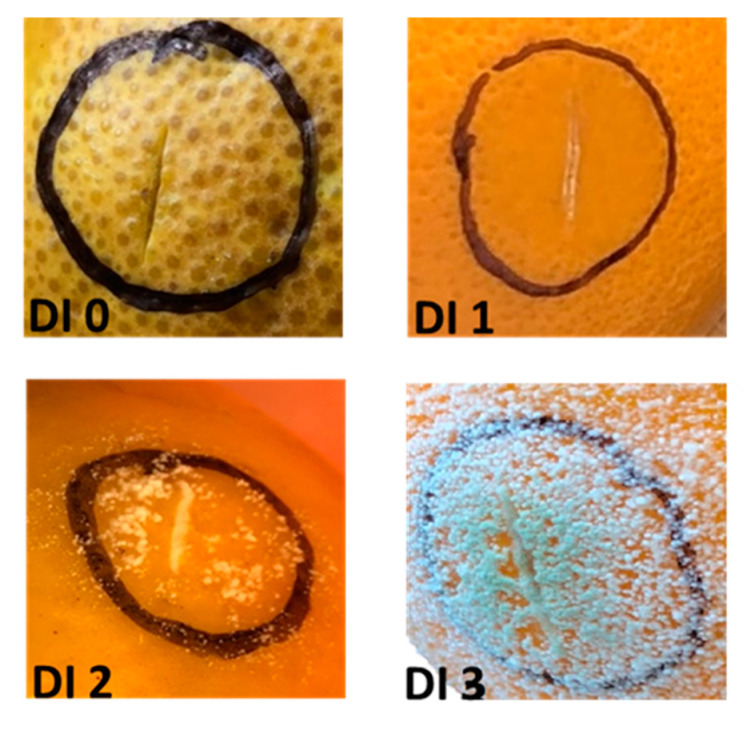
Fruit appearance five days post-inoculation and evaluation of disease index (DI): DI 0 = no symptoms; DI 1 = presence of rot; DI 2 = presence of mycelium; DI 3 = presence of spores.

The development of green mold and the severity of the symptoms were assessed as described in Material and method. Fruits inoculated with water were symptomless at both observation dates. Fruits inoculated with *P. digitatum* showed symptoms after the fifth day from the inoculation. The symptoms consisted of rots which broaden from the point of inoculation to about one centimeter. Most of the inoculation points (91%) showed symptoms and the average disease index at 5 DPI varied from 0.75 to 2.75 (data not shown). The majority of the inoculation sites showed the presence of fungal efflorescence even beyond the area affected by rots (DI 2). In some cases, a slight green color has been observed which indicates that sporulation occurred (DI 3) (Figure 1).

### 2.2. Effect of Fungal Inoculation upon the Anthocyanin Content

**Figure 2:**
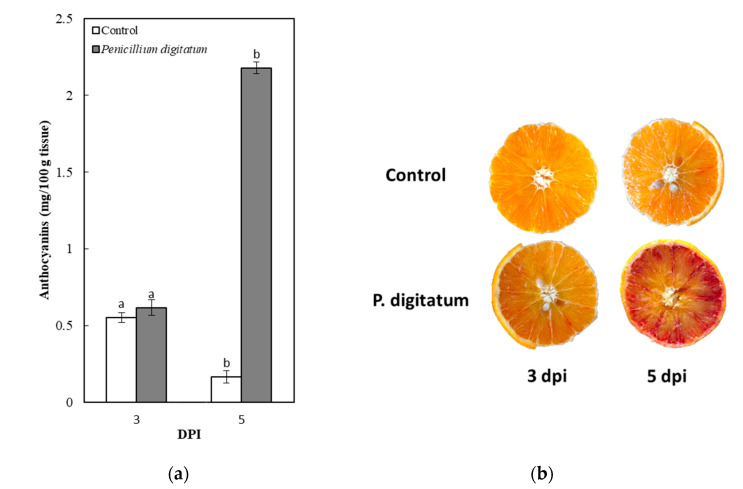
(**a**) Anthocyanin content in control and inoculated fruits; (**b**) Picture of sweet orange control and inoculated fruits. Each bar represents the mean value of three replications ± SD. Significantly different values, within each sampling time, are indicated by different letters (*p* < 0.05).

In Figure 2, the anthocyanin content of both control and inoculated fruits is reported. After 3 days from inoculation (3 DPI), the pigmentation between the control group, represented by the oranges inoculated with H_2_O, and the oranges inoculated with *P. digitatum*, did not show significant phenotypic differences (Figure 2). However, a sharp increase in anthocyanin content occurred 5 days after inoculation (5 DPI) in the infected fruits compared with the control group. At this stage, the anthocyanin level of the inoculated fruits was more than 12 times higher than the control samples (Figure 2) thus indicating that fungal infection induces anthocyanin biosynthesis and causes their content to increase.

### 2.3. Analysis of the Expression of Genes Involved in Anthocyanin Biosynthesis

**Figure 3:**
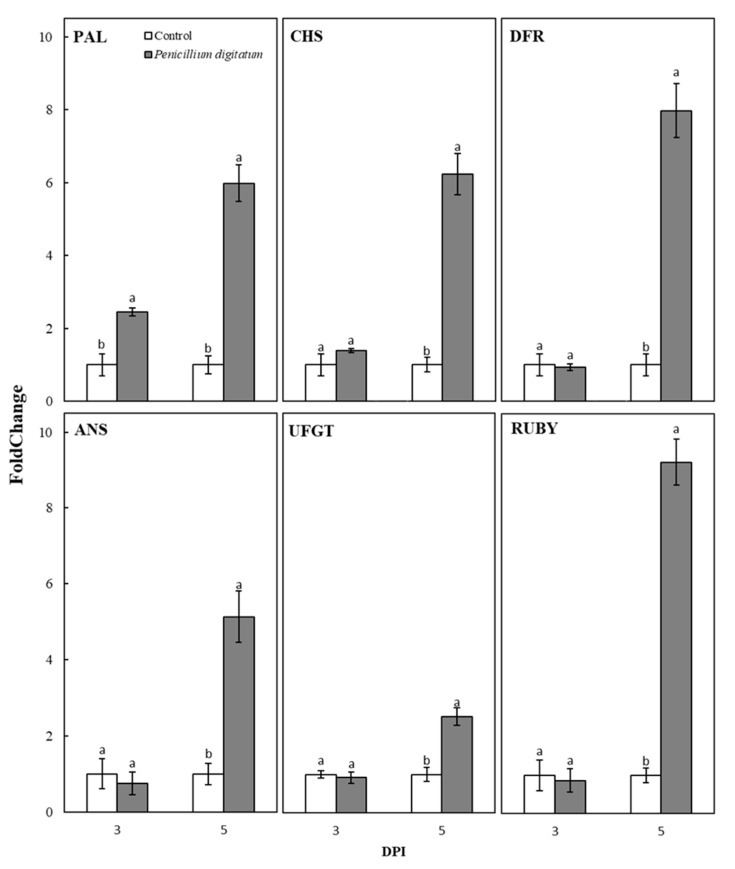
Evaluation of gene expression in control and inoculated fruits PAL, CHS, DFR, ANS, UFGT, and RUBY. Each point represents the mean value of three replications ± SD. Significantly different values, within each sampling time, are indicated by different letters (*p* < 0.05).

Figure 3 reports the relative transcript levels of considered genes using the 2^−∆∆CT^ approach. As shown in Figure 3, the expression of PAL increased in the inoculated fruits from the 3 DPI on, reaching a maximum after 5 days (5 DPI) at which the expression level was 6 times higher than the control fruits. Similarly, the expression of CHS, DFR, ANS, and UFGT increased up at 5 DPI reaching values ranging between 2.5 and 8 times higher than the control fruits (Figure 3). Consequently, the observed enriched pigmentation in the inoculated fruits (Figure 2) is provoked by the induction of the structural genes involved in pigment biosynthesis (Figure 3). In addition, the expression of RUBY markedly increased in inoculated fruits, reaching 5 DPI a value 9 times higher than control fruits, thus indicating that also the main gene-regulating anthocyanin production is activated by the fungus.

### 2.4. DNA Methylation Level of Dfr and Ruby Promoter Regions

**Figure 4:**
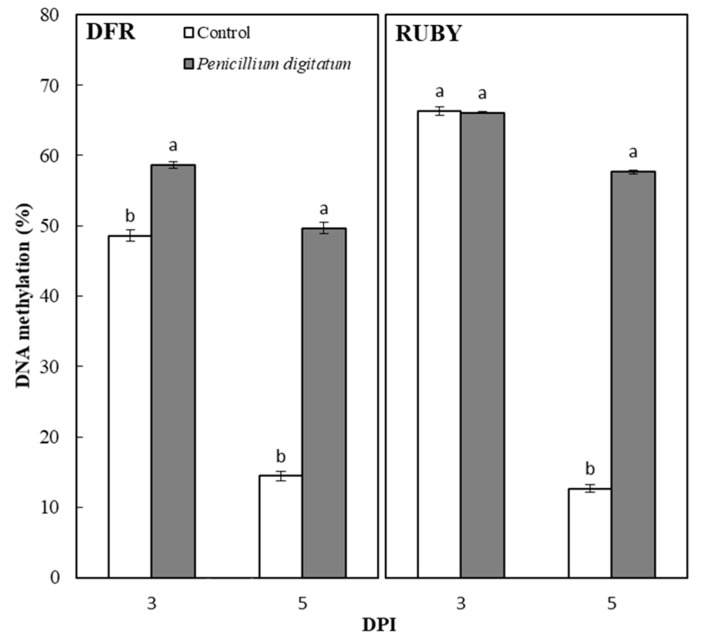
Analysis of the methylation status of both DFR and Ruby promoter regions. Each point represents the mean value of three replications ± SD. Significantly different values, within each sampling time, are indicated by different letters (*p* < 0.05).

The analysis of change in DNA methylation of the DFR and RUBY regions was performed by McrBC digestion followed by real-time fragment quantification as described in the “Materials and Methods” section. Figure 4, shows that the degree of cytosine methylation in the DFR promoter is slightly greater in the inoculated fruits than in the control sample at 3 DPI. However, at 5 DPI, the methylation level of the inoculated fruits exhibited a value of 50% of methylation whereas it sharply decreases in the control fruits showing a value of about 15%. Similarly, the methylation level of the RUBY promoter showed the same methylation percentage (66%) both in the control and in the inoculated fruits at the 3 DPI. By contrast, the RUBY promoter maintained most of the sites methylated (55%) in the inoculated fruits whereas the control fruits registered a dramatic decrease in the methylation percentage (12%), thus suggesting that more extensive demethylation processes occurred in both DFR and RUBY promoter in the control fruits.

### 2.5. Analysis of the Expression of Genes Involved in DNA Demethylation

**Figure 5:**
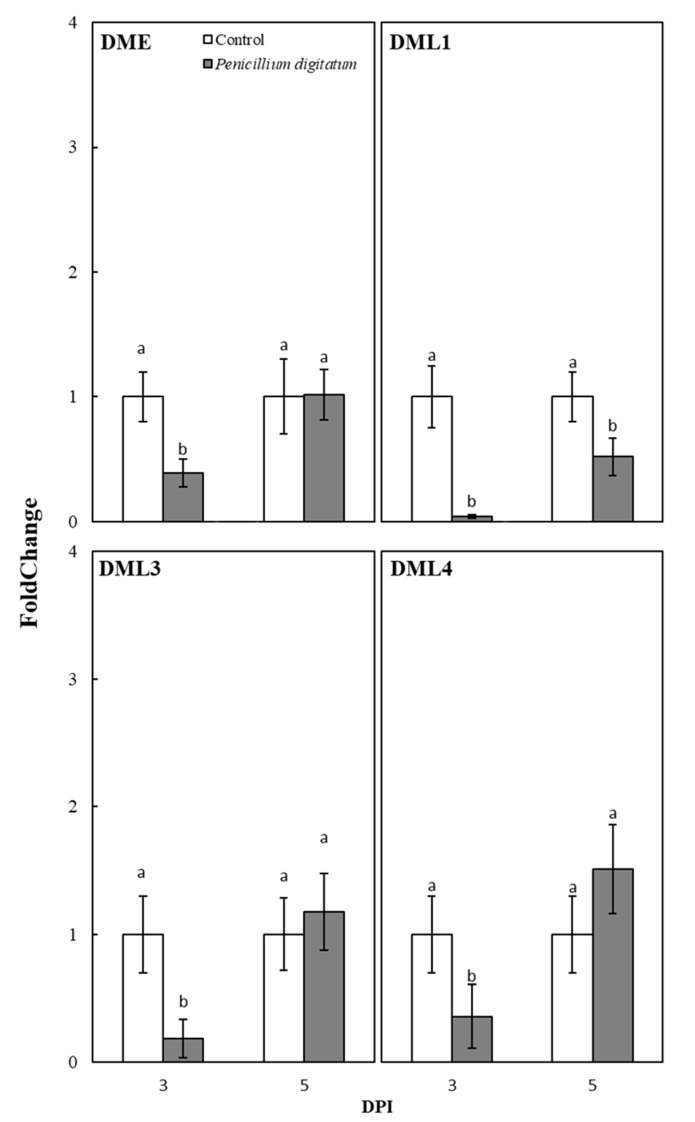
Expression pattern of demethylase encoding genes DME, DML1, DML3, and DML4 in both inoculated and control plants. Each bar represents the mean value of three replications ± SD. Significantly different values, within each sampling time, are indicated by different letters (*p* < 0.05).

As shown in Figure 4, the methylation levels of the DFR and Ruby promoters decreased from 3 DPI in the control samples. The aforementioned decrease bestows a crucial role to DNA demethylases in DNA methylation reprogramming. DNA demethylation in *Arabidopsis* is initiated by 5′-methylcytosine DNA glycosylase/lyase enzymes, including REPRESSOR OF SILENCING 1 (ROS1), DEMETER (DME), DEMETER-LIKE 2 (DML2), and DML3 [23]. Four *At*ROS1 orthologs have been identified in the sweet orange genome [24,25]. They include *Cs*DME, *Cs*DML1, *Cs*DML3, and *Cs*DML4. As shown in Figure 5, the expression level of all de-methylases was repressed by fungal inoculation at 3 DPI. The expression of DML1 was down-regulated in the inoculated fruits also at 5 DPI (Figure 5). The expression level of DME, DML3, and DML4 in the inoculated fruits was comparable with that of control samples at 5 DPI (Figure 5). At this sampling date, the differences of the relative expression values were not statistically significant, suggesting that these genes express similarly at this sampling stage in both control and inoculated fruits.

## 3. Discussion

Sweet orange is a popular fruit in many parts of the world due to its unique taste and flavor. It is well known that a range of environmental factors can enhance anthocyanin content in blood oranges, but the effect of biotic stress upon pigment content is still unknown. Consequently, this study aimed to evaluate the effect of *P. digitatum* inoculations upon both anthocyanin content and the expression of genes implicated in their biosynthesis. The results clearly indicate that anthocyanin content sharply increases in the inoculated fruits and this rise occurs simultaneously with the induction of PAL, CHS, DFR, ANS, UFGT, and RUBY expression. For the first time, these findings correlate pathogen attack directly with anthocyanin biosynthesis in sweet orange. Indeed, it has been previously shown that salicylic acid (SA) applications significantly decrease the postharvest decay of fruits induced by *P. digitatum* as well as positively influence fruit quality parameters such as anthocyanin levels, and the increased amount of anthocyanin was closely related to the antifungal activity triggered by SA in blood oranges [9]. However, as the effect of salicylic acid was not investigated in healthy, not inoculated fruits, it is not possible to exclude that salicylic acid induces anthocyanin enhancement also in the absence of fungus inoculations. Studies have been conducted to identify key genes and proteins induced by *P. digitatum* in not pigmented *Citrus sinensis* fruits such as Navelate and Jincheng varieties thus highlighting key pathways and processes that are influenced; in particular, the biosynthesis of the phenylpropanoid pathway and the expression of phenylalanine ammonia-lyase (PAL) gene were induced [21,22]. PAL is located upstream in the metabolic pathway leading to anthocyanins and is involved in the synthesis of various compounds induced by biotic and abiotic stress. This indicates that a common response to biotic stress is triggered in *Citrus* fruits although the route in blood varieties leads to anthocyanin production via the activation of genes located far downstream the step catalyzed by PAL in the pathway. Epigenetic changes play a pivotal role in inducing plant resistance responses by controlling the expression level of the defense genes [19]. The changes of DNA methylation during fruit development and ripening have been investigated in the fruits of several species, including both climacteric and non-climacteric fleshy fruits [24,26,27,28]. It has been shown that DNA methylation decreased during tomato fruit ripening [26,27]. Active DNA methylation rearrangement is also involved in the development and ripening of sweet oranges in which DNA methylation gradually increased from immature to ripe fruit [24]. These studies suggest that either an increase or a decrease in DNA methylation might be responsible for the normal ripening process. Therefore, the correlation between DNA methylation and gene expression is very variable depending on various factors such as the genomic region, either gene body or promoters, in which the methylation rearrangement occurs [19]. Normally, low levels of DNA methylation on promoter regions result in the activation of gene expression. De-methylation of promoters caused by abiotic stress, such as cold, salinity, and drought, results in upregulation of abiotic stress response genes [25,29,30]. In this work, we evaluated the rearrangement of the methylation level of DFR and RUBY promoters; the selection was made taking into account that both genes hold a primary role in the anthocyanin biosynthesis pathway, their expression being finely regulated in sweet orange [4,7]. Our results indicated that these genomic regions maintained high levels of cytosine methylation in the inoculated fruits compared to homologous regions of the untreated samples. The downregulation of demethylase gene expression observed in correspondence of the 3 DPI in inoculated samples suggests the importance that might be assumed by these genes in the reformulation of the DNA methylation. Moreover, the global analysis of our findings suggests that the induction of genes involved in anthocyanin biosynthesis in the inoculated fruits is accompanied by minor levels of gene demethylation in their promoter regions. This is contrasting with a major part of the results including those regarding the direct correlation between promoter demethylation and gene expression of blood oranges subjected to cold stress [25]. However, it has been shown that hypomethylation in promoters is not always necessary for increased levels of gene expression. In rice, two promoter critical regions of the *Pib* blast resistance gene were heavily CG cytosine-methylated [31]. The induced expression of *Pib* by *M. grisea* infection did not entail its promoter demethylation suggesting that promoter DNA methylation plays an enhancing role in conditioning the high-level of induced expression of the *Pib* gene under *M. grisea* infection [31]. Hypo- and hypermethylation can represent both beneficial strategies to plants under stress conditions, considering that high levels of global methylation will decrease energy consumption by limiting gene expression, whereas demethylation of particular genes, such as defense genes, will enhance their expression to promptly respond to environmental challenges. Nevertheless, the use of McrBC assay might not be exhaustive in defining the total DNA methylation status of the genes, either in the promoters or in the gene body. Future experiments have been programmed that consider a wider analysis of DNA methylation using bisulfite sequencing. Moreover, the occurrence of different mechanisms of gene regulation at the post-transcriptional level cannot be excluded. Indeed, numerous examples are demonstrating the mechanism by which RNA-binding proteins (RBPs) can sense biotic and abiotic signals and carry out a variety of molecular processes on their target RNAs to elicit appropriate responses [32]. 

In conclusion, this is the first report that correlates fungal infection with anthocyanin biosynthesis induction in blood oranges. Moreover, the maintenance of DNA methylation of *dfr* and *ruby* promoters in inoculated fruits is associated with the activation of gene expression. Our study lays the foundation for future work aimed to unravel the role of anthocyanin in protecting sweet orange from disease by comparing their fungal resistance with that of blond oranges in the same conditions.

## 4. Materials and Methods 

### 4.1. Fungal Inoculum Preparation

*P. digitatum* strain MPVCTBP1 was isolated from a green mold naturally infected sweet orange fruit. The isolate was grown and maintained on potato dextrose agar (PDA, Oxoid, Milano, Italy) medium. *Penicillium* mycelium and conidia were recovered by flooding a 5 days old culture dish with sterile water. The resulting suspension was filtered through sterile gauze to remove the mycelium and then centrifuged at 3000 RPM × 15 min to recover conidia that were washed three times with sterile distilled water, according to a slight modification of the method described in Deng et al. [20]. The conidia were resuspended in sterile distilled water and the concentration was measured spectrophotometrically and adjusted to 1 × 10^6^ conidia per milliliter. 

### 4.2. Fruit Material and Inoculation

Red oranges (Tarocco Tapi) [*C. sinensis* (L.) Osbeck] were harvested in January 2019 trees grown at the experimental agricultural field of the University of Catania (Italy). The inoculations were performed according to Platania et al. with minor modifications [33]. Freshly harvested oranges showing no physical injuries or infections were washed with sterile water and successively soaked in 2% sodium hypochlorite for 2 min. Then, they have been washed again with sterile water and allowed to dry for 12 h at room temperature. For inoculations, four wounds (2 mm wide and 4 mm deep), made by paying attention not to go beyond the albedo layer, were made on the opposite sides of two perpendicular lines outlined by placing the fruit stalk upward. *P. digitatum* was inoculated into 6 fruits by pipetting 10 μL of the spore suspension into each wound (24 inoculations for each thesis). Similarly, fruits pipetted with 10 μL of sterile distilled water represented the control samples. Following inoculation, the fruits were individually transferred into a small bowl and covered by the transparent film and incubated at 20 °C, 90–95% relative humidity. Samplings were performed at the 3rd and 5th days after inoculation (DPI) from both wounded (water-injected) and inoculated fruits. During each sampling, 3 fruits were collected, and their flesh was mixed to generate three replicates (1 g each) of an average sample. Then, the orange flesh was immediately frozen with liquid nitrogen and stored at –80 °C until used. The development of green mold and the severity of the symptoms were assessed at each inoculation point three- and five-days post-inoculation by a disease index (DI) scale based on four values: DI 0 = no symptoms; DI 1 = presence of rot; DI 2 = presence of mycelium; DI 3 = presence of spores [33].

### 4.3. Extraction of Total RNA and cDNA Synthesis

The total RNA from orange fruit flesh and reverse transcription were conducted as described in Sicilia et al. 2020 [25]. RNA samples without reverse transcriptase were routinely included as a negative control.

### 4.4. Measurement of Gene Expression

Real-time qRT-PCR was performed with PowerUp SYBR Green Master mix by ThermoFisher Scientific and carried out in the Bio-Rad iQ5 Thermal Cycler detection system. The relative quantitation of expression of genes involved in anthocyanin biosynthesis and regulation (PAL, CHS, DFR, ANS, UFGT, and RUBY), and in DNA demethylation (DME, DML1, DML3, and DML4) was performed as detailed in Lo Piero et al. [10]. Primer sequences are shown in Table S1.

### 4.5. Total Anthocyanins Content

Anthocyanin determination was performed according to the method described in Lo Cicero et al. [34].

### 4.6. Methylation Sensitive Digestion

DNA extraction and the evaluation of quality and integrity parameters were performed as described in Sicilia et al. 2020 [25]. DNA digestion was carried out using the methylation-sensitive endonuclease McrBC (New England Biolab Inc., Ipswich, MA, USA) according to the manufacturer protocol [35] followed by enzyme inactivation at 65 °C for 20 min. As McrBC requires GTP for cleavage [36], for each sample, both a reaction in which GTP is included in the digestion mix and a reaction in which GTP is excluded were prepared. Real-time PCR was performed as described in Sicilia et al. 2020 [25]. Primer sequences are showed in Figure S2 and listed in Table S1 (indicated by the wording “*pro*”). 

### 4.7. Statistical Analysis

Data were analyzed by one-way ANOVA (*p* < 0.05) followed by Tukey’s test for multiple comparison procedures using the statistical software package Statistica v. 13.0 (Dell Inc., Round Rock, TX, USA).

## Data Availability

Data is contained within the article or supplementary material.

## Supplementary Materials

The following are available online at https://www.mdpi.com/2223-7747/10/2/244/s1, Figure S1: Scheme of the anthocyanin biosynthesis pathway, Figure S2: DFR and RUBY promoter sequences, Table S1: Primer sequences and real-time PCR conditions.
