# Supplementary material for: Fungal Infection Induces Anthocyanin Biosynthesis and Changes in DNA Methylation Configuration of Blood Orange [Citrus sinensis L. (Osbeck)]
Source: Plants (Basel). 2021 Jan 27;10(2):244. doi: 10.3390/plants10020244 (PMC7910907; doi:10.3390/plants10020244)
Supplement: Supplementary file 1 [file plants-10-00244-s001.zip › Table S1.docx]

Table S1- Primer sequences and real time PCR conditions.

| Primer | Sequence | TM (°C) | Amplicon's size (bp) | Gene's accession | Database |
| --- | --- | --- | --- | --- | --- |
| Cs_CHS_For | 5’-TCTATGGACGGGCATCTTC-3’ | 58 | 102 | AB009350.1 | NCBI |
| Cs_CHS_Rev | 5’-TGCCTCGGTTAGGCTTTTC-3’ | 58 |  |  |  |
| Cs_DFR_For | 5’-GCTGTTCGTGCTACTGTTC-3’ | 58 | 108 | AY519363 | NCBI |
| Cs_DFR_Rev | 5’-GGCTAAATCGGCTTTCCATA-3’ | 58 |  |  |  |
| Cs_ANS_For | 5’-GGTGACTGCTAAATGTGTT-3’ | 58 | 104 | AY581048.1 | NCBI |
| Cs_ANS_Rev | 5’-CAAGTCCCCTGTGAAGAATA-3’ | 58 |  |  |  |
| Cs_UFGT_For | 5’-TCTTCAGCACTCCGCAATC-3’ | 58 | 95 | NM_001320060.1 | NCBI |
| Cs_UFGT_Rev | 5’-TCCATCGGATACGTCGTAAG-3’ | 58 |  |  |  |
| Cs_Ruby_For | 5'-ACAATCCACCCCGTCTGATC-3' | 59,4 | 227 | JN402330.1 | NCBI |
| Cs_Ruby_Rev | 5'-CTGGCCTGCTTCAATGACTC-3' | 59,4 |  |  |  |
| Cs_DME_For | 5’-CAGAAACCGCCCAAACGAAG-3’ | 60 | 185 | orange1.1t01511.1_Hzau_Valencia_v2.0 | www.citrusgenomedb.org |
| Cs_DME_Rev | 5’-GCATCGGTTGTCTCCCTGAT-3’ | 59,8 |  |  |  |
| Cs_DML1_For | 5’-GCCGCAGAATCCACTAACCT-3’ | 60 | 181 | Cs6g15500.2_Hzau_Valencia_v2.0 | www.citrusgenomedb.org |
| Cs_DML1_Rev | 5’-CTTTACACAGCTGCCCGGTA-3’ | 60 |  |  |  |
| Cs_DML3_For | 5’-CGGCGAAAAAGCAACTCCAA-3’ | 60 | 164 | Cs3g07800.1_Hzau_Valencia_v2.0 | www.citrusgenomedb.org |
| Cs_DML3_Rev | 5’-CTATGGTTCTGCCAGCGACA-3’ | 60 |  |  |  |
| Cs_DML4_For | 5’-GAAACCAGGCAAGACCCGTA-3’ | 60 | 176 | Cs5g04950.1_Hzau_Valencia_v2.0 | www.citrusgenomedb.org |
| Cs_DML4_Rev | 5’-TTCATATCCAACGGCACGCT-3’ | 60 |  |  |  |
| Cs_Ruby_Pro2_For | 5'-CGATGGAGTTTGGGCTTGAG-3' | 59,4 | 241 | JN402330.1 | NCBI |
| Cs_Ruby_Pro2_Rev | 5'-CCAGTCCAAGTTAACAATTCCCA-3' | 58,9 |  |  |  |
| Cs_DFR_pro_For | 5'-ACCCAAAAGTAGGCCCAAGT-3' | 57,3 | 198 | scaffold00002 | www.citrusgenomedb.org |
| Cs_DFR_pro_Rev | 5'-GTTGCCGGGCTTGTTTATGT-3' | 57,3 |  |  |  |

| Amplification program | | |
| --- | --- | --- |
| Temperature | Time | N° of cycles |
| 50° C | 2’ | 1 |
| 95° C | 2’ | 1 |
| 95° C | 15’’ | 40 |
| 2° C lower than Tm | 15’’ |  |
| 72° C | 1’ |  |
| Melting curve | | |
| 95° C | 15’’ | 1 |
| 55° C | 1’ | 1 |
| 55° C (+0.5°C/cycle) | 15’’ | 80 |
