# Supplementary material for: Fungal Infection Induces Anthocyanin Biosynthesis and Changes in DNA Methylation Configuration of Blood Orange [Citrus sinensis L. (Osbeck)]
Source: Plants (Basel). 2021 Jan 27;10(2):244. doi: 10.3390/plants10020244 (PMC7910907; doi:10.3390/plants10020244)
Supplement: Supplementary file 1 [file plants-10-00244-s001.zip › Figure S2.docx]

DFR promoter sequence

CTGAAAAGCAGCGATTCAAAATTTTCAAGTCACAGTAACTAGTATTTATCAAAATTTTCGAATCATAATAACAAATATTTATCACAAACACTTTAATATTGTAACTTAAACGTTACAGTTCTCTAATCTCAATACTAAGTATGTTTTTACAATATAAGTTTGAATCAATTTTTTTTAGTTTTCACTTCTATAGCCTAATAATCATTGATTCAATACTCAACAAAAGATAAGTTTAATATTGAATTATTGTCTACTCAACTAACTTGTACACTTATCTCTTATCTACATTAATTTGAGAGATAATCTCACAAGTAAATGATAGAACTACAAATTATTTTTGATAATTTAATGTGGCATAATCATTTTGGTTAGATGAACAAAAATATTCTTAAACGGCATCGATATTTACGAAAAGTTTGTGAATAATACAAACTCTCTTTGATTCAATAGCTCGCTCTTTTATCTAGCTCTTCCGCAAGTTTTGCAAGTAAGGCCTTATTCTTTTTGTTATTAAGATCGAAGGACTGAACTTCCGATGTGATCAGAATCATGATTGGGCGTTCATAAGAATGACAAGTAAAGGAAATTGGGTTGTTGTGCTTCATAATAATAAAGGACGTTTGTGTTAGAATTAGTGGGGAAACGAGTTTGGAATTTACGTGTGCTGCAAGATTTTCTCCCTAATAGTAACACAAGCAACCCAAAAGTAGGCCCAAGTCATATACCGTCCGTCGAGGGTCAAATATTTTCGGCATGTGGGCAGATGACAACTACCACGCACGTGCAAAAACGGTCAGTTGATCACGTGGAGGGTAATTGGAGCTAAAAGGGGGCGTGTTAGTCTGTTAGATGAAACTACAACATCTACTATTATATACATAAACAAGCCCGGCAACGAATGAACTTCAACTGCAAGAGTTTCGAAATTCCTTGATCAAACATTTAGTATCAGTAGTCCGAATTTTTTTTTTTTTAAAAAAAAGGTCATG

Ruby promoter sequence

gagagtataccgtatgcgtacacatcaatattgatactagctagatagctaggttggtccctggctatagctattaaaaaaaaaaaaagttatgtgcttaattattgccaccaaaagagtcattgggctggaagttagttggagaaaatattgtacagaaaaaaaaaaaaaaaacgatggagtttgggcttgagttctcattgcgtccccttggcgggaagcactgtacaagaactttaggccgttcaaaactttaattatcacacggcatttttttttttttttttaatagctgtagccagggacatctggagtttgatgaaagacactcaatattcaacagataagcattagacgcacttgttttttctgtttcaacttgttaatggttttgggaattgttaacttggactggtagttgtaattaactgtcaaagtcggtggccacaaggcaaattcaagttggctttgacaagccggccaaagaaaatggtgcagccggccaaagaaaatggtgccgtccaaatggagaaaaagaagaagttggcaacaacatttgaaatatgtatatattattttaattaccattttttggctataaaagggagaactccctaattcatttatcatccaattttgtagagagaattgagagttgtgagaagtgattcttgcaagagcaaagaattttgtgtgcttagtgatttgagagtttgggtgtattggggttttgggtagtgagctaaaatactacaatacttgtaactccttttcactagtataatatttctttctgtcttcgcccgtggacgtaggctaaaagccgaaccacgtaatttctggtattttcttttgtgcttgttctttatttttctatcaattttactttagctgcgtgtctgcttcacccaccaatttcctaacattaacaagtattgtttactatttttggacgaagaatagtagaagtagtttccttgtggatgcaagacaagcacgtcactctctccgaaaaggcttaattgatcgacgtagcatgaagtgaggagcacgtattattatacaagcagctgttctgtaggctctttaaattttataaaaaaagagagttgagtaagtgtaggtgctaattaaattttgattttttaggtaagcacatatactacacatagggtctttatg

**Figure S2 – DFR and RUBY promoter sequences.** In yellow forward and reverse primers, in green the first ATG (start codon).
