# Supplementary figures and images for: Fungal Infection Induces Anthocyanin Biosynthesis and Changes in DNA Methylation Configuration of Blood Orange [Citrus sinensis L. (Osbeck)]
Source: Plants (Basel). 2021 Jan 27;10(2):244. doi: 10.3390/plants10020244 (PMC7910907; doi:10.3390/plants10020244)

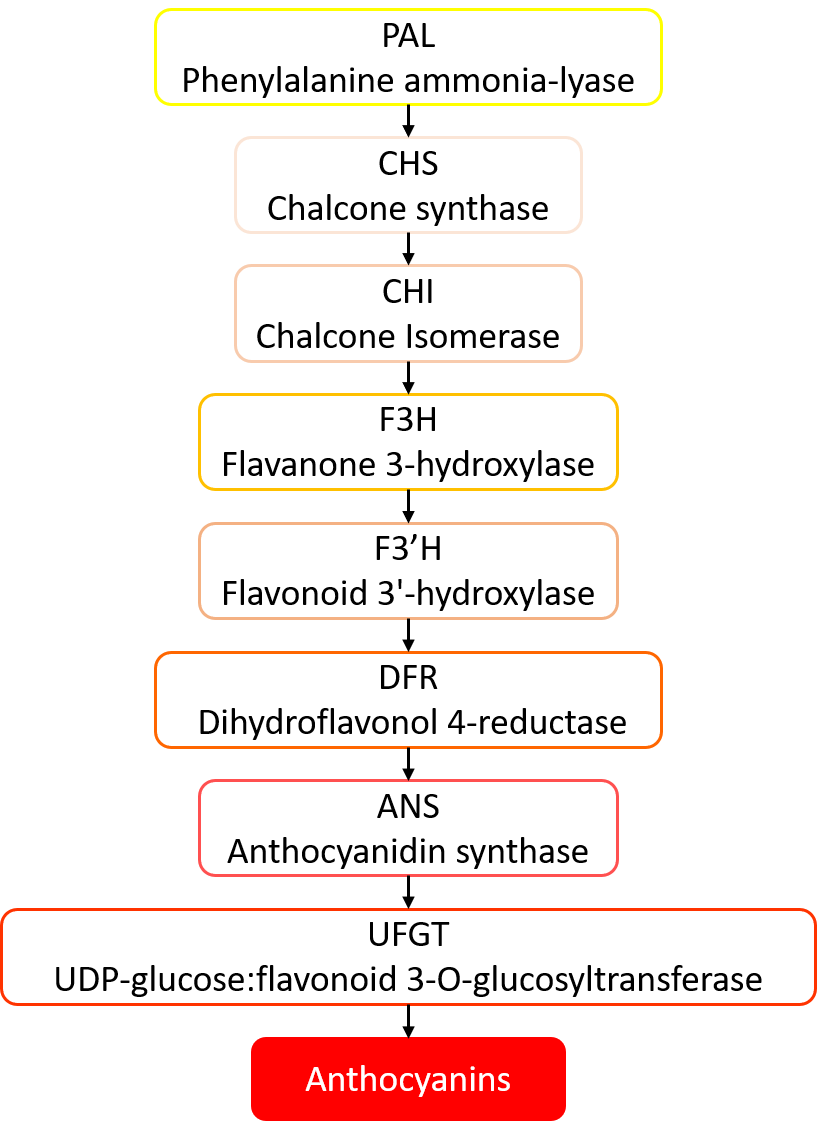


**Figure S2**: Scheme of the anthocyanin biosynthesis pathway

Supplement: Supplementary file 1 [file plants-10-00244-s001.zip › Figure S1.docx]
